# A workshop report on HIV mHealth synergy and strategy meeting to review emerging evidence-based mHealth interventions and develop a framework for scale-up of these interventions

**Published:** 2011-11-11

**Authors:** Sarah Karanja, Lawrence Mbuagbaw, Paul Ritvo, Judith Law, Catherine Kyobutungi, Graham Reid, Ravi Ram, Benson Estambale, Richard Lester

**Affiliations:** 1 Department of Medical Microbiology, University of Manitoba, Nairobi, Kenya; 2 Centre for the Development of Best Practices in Health, Yaoundé Central Hospital, Yaoundé, Cameroon; 3 School of Kinesiology and Health Sciences, Department of Psychology, York University, Ontario, Canada; 4 Division of Infectious Diseases, University of British Columbia, Vancouver, Canada; 5 African Population Health and Research Center, Nairobi, Kenya; 6 African Medical and Research Foundation, Nairobi, Kenya; 7 Institute of Tropical and Infectious Diseases, University of Nairobi, Nairobi, Kenya

**Keywords:** mHealth, mobile phones, anti-retroviral therapy, adherence, retention

## Abstract

mHealth is a term used to refer to mobile technologies such as personal digital assistants and mobile phones for healthcare. mHealth initiatives to support care and treatment of patients are emerging globally and this workshop brought together researchers, policy makers, information, communication and technology programmers, academics and civil society representatives for one and a half days synergy meeting in Kenya to review regional evidence based mHealth research for HIV care and treatment, review mHealth technologies for adherence and retention interventions in anti-retroviral therapy (ART) programs and develop a framework for scale up of evidence based mHealth interventions. The workshop was held in May 2011 in Nairobi, Kenya and was funded by the Canadian Global Health Research Initiatives (GHRI) and the US Centre for Disease Control and Prevention (CDC). At the end of the workshop participants came up with a framework to guide mHealth initiatives in the region and a plan to work together in scaling up evidence based mHealth interventions. The participants acknowledged the importance of the meeting in setting the pace for strengthening and coordinating mHealth initiatives and unanimously agreed to hold a follow up meeting after three months.

## Introduction

There have been calls for more evidence on the effectiveness of mHealth interventions to improve health outcomes. The WelTel Kenya1 intervention evaluating the use of mobile phones to improve adherence to ART is the first randomized controlled trial to show the effectiveness of using mobile phones for viral suppression [1]. With these findings in place and with the ever-increasing mHealth interventions being developed, a coordinated approach among various players is essential to maximize the potential of mHealth in health system strengthening. This was the rationale for a meeting of mHealth stakeholders that reviewed the work that is going on in the region and aimed at forging a united front for scaling up evidence-based mHealth interventions. The two-day workshop in May 2011 was held in Nairobi, Kenya and was jointly organized by the University of British Columbia (UBC) and the University of Nairobi Institute of Tropical and Infectious Diseases (UNITID), and funded by Global Health Research Initiative (GHRI) and Centre for Disease Control and Prevention (CDC) Kenya.

High level adherence of 95% and above that can yield maximum viral suppression and reduce drug resistance to antiretroviral medications is still a challenge. Earlier studies with meta-analyses on adherence to anti-retroviral therapy (ART) in sub-Saharan Africa found that a pooled estimate of 77% of patients in African settings achieved adequate adherence (95% of prescribed pills), compared with 55% of patients in North American settings [2]. However, adherence still poses a challenge in resource limited settings and there is still evidence of high rates of patient lost-to-followup[3].

Interventions using mobile phones to improve health outcomes such as adherence and retention in the ART programs are likely to be viable due to the wide access to mobile phones in most developing nations [4], the emerging evidence to support use of mobile phone interventions to improve health service delivery and the likelihood of these interventions leading to cost saving measures in the health system.

## Workshop report

### Aim of the workshop

The aim of the workshop was to bring together various stakeholders who included researchers, academicians, information, communication and technology programmers, policy makers and members from the civil society to:1) review evidence based mHealth research for HIV care and treatment; 2) review mHealth applications developed to support adherence and retention in ART programs; 3) develop an mHealth evidence based framework for scale up and 4) identify gaps in evidence and the areas where more research is needed.

On the first day of the workshop the mHealth concept, ART adherence and retention challenges and the role of mHealth, evidence for HIV-related mHealth interventions and mHealth interventions in other areas were presented and discussed. The second day of the workshop was dedicated to building partnerships and developing a framework for mHealth strengthening to support HIV care and treatment and other health conditions.

### Participants

Fifty participants from twenty nine organizations in Kenya, Uganda, Zambia, Cameroon and Canada attended the workshop. They included researchers, policy makers, health care implementers, information, communication and technology programmers, academicians and civil society representatives. Although the workshop initially targeted thirty participants, the high demand necessitated a request for additional funding to accommodate more participants.

### Day 1: mHealth Projects and lessons learned

There were seventeen brief presentations during the first day, in which thirteen mHealth projects either in progress or completed, were discussed (Table 1 and Table 2). Some of the key issues and lessons learned from these presentations included:

**Table 1:** mHealth projects presented during the HIV mHealth synergy and synthesis workshop in Nairobi, Kenya, 23-24 May 2011

Project	Organization	Country	Activities
WelTel Kenya1 (Final Results)	WelTel, Universities of Manitoba, Nairobi and British Columbia	Kenya	A randomized controlled clinical trial to assess use of text messages to improve antiretroviral medications adherence and viral suppression. The study findings show that patients who received text messages had significantly improved self-reported adherence and viral suppression rates.
PMTCT (Prevention of Mother to Child Transmission) Study (Ongoing)	Universities Manitoba and Nairobi	Kenya	A randomized controlled clinical trial to assess use of text messages to Improve ante natal clinic visits, increase usage of Nevirapine and decrease number of HIV infected infants born to HIV positive mothers. Study ongoing.
CAMPS(Cameroon Mobile Phone SMS) Trial (Ongoing)	Centre for the Development of Best Practices in Health, Yaoundé	Cameroon	A randomized controlled single blinded trial to test the efficacy of text messages and motivational messages in improving adherence to antiretroviral medications. Study ongoing.
Programmatic evaluation of Cell phone use as an intervention for antiretroviral resistance	KEMRI (Kenya Medical Research Institute)	Kenya	A pilot study to determine perceptions regarding use of cell phone to support treatment adherence among people living with HIV. Patients prefer to use mobile phones to facilitate access to care than to receive medication reminders.
SMS Printer	Ministry of Health, Zambia	Zambia	A pilot study to ascertain whether use of text message technology can reduce laboratory results turn-around time. Turn- around time was reduced to about 2.6weeks from baseline >6weeks
Better Health Outcomes through Health Mentoring (BHOMA)	Ministry of Health, Zambia	Zambia	A pilot study to evaluate use of text messages to prompt mentored lay workers to trace patients with health problems and refer them to the health facility.
HIV Track SMS Reminder System	APHIA Plus Western Kenya	Kenya	A program that will use text messages as a reminder tool for clinic appointment and drug refill.
Mobile phones for ART service provision	AMREF	Kenya	Use of mobile phones by health care providers as a way to disseminate or access challenging information on HIV care and treatment.
IQSMS for PMTCT	Futures	Tanzania	Use of text messages to support PMTCT data reporting.
Emit (Evaluating and Monitoring Information System)	Cell life/KETAM	South Africa	Use mobile phones for data collection, monitoring and evaluation.
Cellphones4HIV	Cell life/KETAM	South Africa	Use of mobile phone technology in health related mass messaging.
Use of smart phones to monitor type 2 diabetes	York University	Canada	Use of smart phones to improve blood glucose control in type 2 diabetic patients.
Use of smart phones to monitor heart failure	York University	Canada	Use of smart phones to remotely monitor weight and blood pressure in heart failure patients.

**Table 2:** Other organizations making presentations

Organization	Activities
NEPHAK	Improving quality of life of people living with HIV through social mobilization, advocacy and capacity building. Key areas identified that can be strengthened using mHealth technology include; receiving information on commodity and drug stock outs and relaying information on human rights violation.
I-TECH	Strengthening health systems through development and implementation of standardized electronic medical records system, interoperability solutions and capacity building. I-TECH is also spearheading the development of standardized guidelines for EMR systems in Kenya.
APHRC	Conducts research in four key areas: education, health, population dynamics and urbanization. The evidence acquired is then used for policy engagement. See a place for mHealth interventions in the management of chronicity.
Makerere University	Makerere University's Clinical Trials Unit presented on evidence from Uganda on strategies to improve adherence and treatment outcomes. These strategies include: treatment assistants, group adherence counseling, home based counseling, nutritional support and provision of transportation cost.
IDRC	Program areas include: enhancing equity and access to health care through evidence based research, funding and facilitating innovation, inter-disciplinary research and training programs, and building capacity to conduct HIV prevention trials. IDRC, through GHRI, is funding the trial on harnessing mobile phones for prevention of mother to child transmission in Kenya.

1) Adherence to antiretroviral medications continues to be a serious problem. Lester et al., 2010 mobile phone adherence intervention conducted in Kenya report that only 66% of patients on ART in the control arm with viral loads available achieved viral suppression (<400 copies/ml) after one year [1]. A study by Eleches et al., 2011 reported that only 40% of the patients achieved adherence levels above 90% over one year [5]. A study in Cameroon reported that 77% of patients on ART had poor adherence to clinical appointments [6]; 2) Evidence on interventions that use of mobile phones to support patients taking antiretroviral medications is emerging and these interventions can be used together with other proven interventions such as targeted adherence counseling to improve adherence; 3) Evidence suggests that weekly text message reminders are more effective in improving adherence compared to daily text messages [1,5]. A survey of patients attending a clinic in Nairobi, Kenya suggested patients are more interested in using cell phones to access health care providers than for medication reminders. Mobile phone initiatives may therefore be most effective when they are designed to link patients to health care providers through improved patient-provider communication that provides regular support to patients. This was also demonstrated by the WelTel Kenya1 trial; 4) Evidence based mHealth interventions to improve health outcomes need to be scaled up to ensure that the impact is felt by a large number of people in order to enable equitable access to health service provision throughout the country; 5) Proven HIV mHealth interventions may be immediately applicable to the management of other diseases requiring long term medication such as tuberculosis, diabetes, asthma and cardiovascular diseases; 6) Development of mHealth applications should be guided by health system needs and not simply driven by the technology; hence the importance of involving all the stakeholders such as health care providers, patients, government and other stakeholders in needs assessment before developing the applications; 7) Engagement of patients is critical in developing mHealth interventions to ensure their values and perceptions are incorporated to achieve the right content (message) and intensity (message dose) of the text messages. Specific considerations that would need to be taken into account for reviews of the existing and upcoming evidence (Table 3) based on ongoing and completed studies [5,7,8] were also identified; 8) There is a potential for mHealth to improve health management information system (HMIS) related to HIV/AIDS and other health interventions in relation to data accuracy, completeness and timely reporting, leading to improvements in ARV stock management in health centres, better access to pharmaceuticals among HIV/AIDS patients, and ultimately higher rates of ARV adherence due to reduced health system failures.

**Table 3:** Considerations for authors reviewing the evidence on mobile phone text messages for improving adherence to ART

Study	Pop-Eleche et al	Mbuagbaw et al*	Lester et al	Da Costa et al*
Location	Kenya	Cameroon	Kenya	India
Timing	Weekly/Daily	Weekly	Weekly	Weekly
Length	Long/Short	Long	Short	NA
Feedback	No	Yes	Yes	No
Content	Motivational	Motivational	Support/Access	Picture
Sending	Automated	Manual	Manual	Automated
Measurement	MEMS	VAS/SR/PRD	SR/VL	VL (TTE)/SR
Variety	No	Yes	No	No
Duration	12 months	6 months	12 months	24 months
Phone provided	Yes	No	No	Yes
Sample size	431	198	538	600

*Based on protocol; SR: Self Report; VL: Viral Load; PRD: Pharmacy Refill Data; TTE: Time-to-event; MEMS: Medication Event Monitoring System

### Day 2: Way forward/ recommendations

The second day of the workshop was dedicated to developing a strategic action plan for strengthening of mHealth interventions to support care and treatment of HIV and other conditions. The participants formed three discussion groups that formulated the following recommendations:

1) To develop and submit a harmonized regional evidence-based ART adherence and retention mHealth intervention. A regional stepped wedge study design [9,10] based on the evidence of the WelTel Kenya1 randomized controlled trial was proposed for regional implementation; 2) To work towards developing policy guidelines on mHealth implementation as well as ensure the existence of a legal framework to guide scale-up of mHealth interventions and uphold confidentiality and privacy of patient data. Participants suggested they would like to be involved in developing mHealth guidelines and since there is an existing e-Health committee in Kenya, one of the participants should be assigned to represent the mHealth pillar in this committee; 3) To work towards interoperability of mHealth systems to facilitate and foster collaboration in development of viable applications that are scalable, open source and can measure health outcomes; 4) To develop and communicate best practices in strategies and approaches for private-public partnerships in mHealth. Participants were interested in understanding how others have created strategic private-public partnerships and how these approaches can be used in mHealth; 5) To organize a follow up mHealth strategy meeting in three months time to concretize the recommendations developed during this meeting. The subsequent meetings should ensure adequate engagement of the government, donor agencies such as WHO, CDC UNAIDS, USAID, and IDRC and pharmaceutical companies.

## Conclusion

The workshop generated greater interest for participation than expected which indicated the desire for a coordinated approach to implementing mHealth interventions in the region. It created a forum for participants to share and review mHealth interventions in the region. The evidence reviewed suggests that mobile phones can be used as monitoring and supporting tools to provide regular communication between patients and health care providers. A regional evidence-based cell phone intervention to support patient adherence and retention in ART programs was proposed. Participants acknowledged the importance of the workshop and agreed to hold a follow up meeting to reinforce the recommendations developed during this workshop.

## References

[1] Lester RT, Ritvo P, Mills EJ, Kariri A, Karanja S, Chung MH (2010). Effects of a mobile phone short message service on antiretroviral treatment adherence in Kenya (WelTel Kenya1): a randomised trial. Lancet..

[2] Mills EJ, Nachega JB, Buchan I, Orbinski J, Attaran A, Singh S (2006). Adherence to antiretroviral therapy in sub-Saharan Africa and North America: a meta-analysis. JAMA: the journal of the American Medical Association..

[3] Rosen S, Fox MP, Gill CJ (2007). Patient retention in antiretroviral therapy programs in sub-Saharan Africa: a systematic review. PLoS medicine..

[4] Kujawaski Mike (2009). Latest mobile phone statistics from Africa and what it means.. http://www.mikekujawski.ca/2009/03/16/latest-mobile-phone-statistics-from-africa-and-what-this-means.

[5] Pop-Eleches C, Thirumurthy H, Habyarimana JP, Zivin JG, Goldstein MP, de Walque D (2011). Mobile phone technologies improve adherence to antiretroviral treatment in a resource-limited setting: a randomized controlled trial of text message reminders. AIDS..

[6] Mosoko JJ, Akam W, Weidle PJ, Brooks JT, Aweh AJ, Kinge TN (2011). Retention in an antiretroviral therapy programme during an era of decreasing drug cost in Limbe, Cameroon. Journal of the International AIDS Society..

[7] Mbuagbaw L, Thabane L, Ongolo-Zogo P, Lester RT, Mills E, Volmink J (2011). The Cameroon mobile phone SMS (CAMPS) trial: a protocol for a randomized controlled trial of mobile phone text messaging versus usual care for improving adherence to highly active anti-retroviral therapy. Trials..

[8] De Costa A, Shet A, Kumarasamy N, Ashorn P, Eriksson B, Bogg L (2010). Design of a randomized trial to evaluate the influence of mobile phone reminders on adherence to first line antiretroviral treatment in South India--the HIVIND study protocol. BMC medical research methodology..

[9] Brown CA, Lilford RJ (2006). The stepped wedge trial design: a systematic review. BMC medical research methodology..

[10] Padian NS, Holmes CB, McCoy SI, Lyerla R, Bouey PD, Goosby EP (2011). Implementation science for the US President's Emergency Plan for AIDS Relief (PEPFAR). J Acquir Immune Defic Syndr..

